# Supplementary material for: Are carbon emissions trading and green financial instruments synergistic? -Comprehensive quantitative research based on content analysis
Source: PLoS One. 2024 Mar 7;19(3):e0298601. doi: 10.1371/journal.pone.0298601 (PMC10919601; doi:10.1371/journal.pone.0298601)
Supplement: S1 Data — (ZIP) [file pone.0298601.s001.zip › Data/地级市Z_F工作报告.zip!/╣π╢1⁄2/║╙╘┤╩╨/└┤╫╘╣1⁄2╓┌║┼í╛┬φ┐╦╩2╛▌═°í┐_2010.docx]

各位代表：

我代表市人民政府向大会作政府工作报告，请予审议，并请各位政协委员和其他列席人员提出意见。

一、2009年工作回顾

2009年，是新世纪以来我市经济社会发展面临困难和挑战最大的一年，也是我市经受严峻考验取得新的可喜成绩的一年。面对国际金融危机的持续冲击，市政府在省委、省政府和市委的正确领导下，在市人大及其常委会和市政协的监督支持下，以科学发展观统领经济社会发展全局，立足保增长、促需求、优结构、增后劲，带领全市人民，变压力为动力，化挑战为机遇，沉着应对，攻坚克难，有效遏止了经济增长明显下滑态势，实现了经济回升向好发展，社会各项事业全面进步，人民生活不断改善，依期完成了市五届人大四次会议确定的目标任务。

——保增长目标依期实现。针对金融危机冲击引起我市中小企业成本上升、订单减少、生产困难以及投资下降、出口减缓、利用外资回落的严峻形势，我市紧紧抓住国家和省出台扩内需、促增长系列措施的有利机遇，果断实施财政金融“双扩张”策略，突出投资驱动，巩固消费、出口，陆续启动了一批事关河源长远发展的重大基础设施、重要产业和民生项目，出台了《关于实施“暖企工程”的意见》等政策文件，积极帮扶企业渡过难关，营造逆势而上的“小气候”，促使国民经济继续保持平稳较快发展势头。全市完成生产总值416.9亿元，增长10.7%，其中第一产业增加值52.5亿元，增长4.9%；第二产业增加值223.9亿元，增长12.2%；第三产业增加值140.5亿元，增长10.4%；三大产业结构由上年的13.1∶54.1∶32.8调整为12.6∶53.7∶33.7。人均生产总值14325元，增长8.2%。在淘汰落后产能减少工业产值50多亿元的情况下，实现全社会工业总产值670.4亿元、工业增加值205.2亿元，分别增长14%和13.5%，其中规模以上工业总产值626.7亿元、工业增加值189.5亿元，分别增长14.8%和14%。地方财政一般预算收入20.18亿元，增长14.6%。税收总收入40.9亿元，增长8.8%。全社会固定资产投资198.1亿元，增长10.1%，增幅提高35个百分点。实际利用外资2.3亿美元，总量居全省第12位。外贸出口14.2亿美元，增长13.3%，增幅居全省第3位。居民消费价格涨幅得到有效控制，全年下降2.4%。年末城镇登记失业率3.07%。城镇居民人均可支配收入12137元，增长7%。农村居民人均纯收入5045元，增长6.7%。人均生产总值、工业增加值、外贸出口、城镇居民可支配收入、农村居民人均纯收入等主要指标提前一年完成了“十一五”规划预期目标。

——“双转移”取得重大成果。市政府和中山（河源）产业转移园在全省产业转移目标责任考核中均被评为优秀。中山（河源）产业转移园再次竞得省政府5亿元竞争性扶持资金。全市“一区六园”完成规模以上工业总产值、工业增加值和入库税收分别为368.8亿元、105.4亿元和7.46亿元，分别增长6.8%、4%和15.4%，占全市规模以上工业总量近60%，成为拉动全市经济发展的重要增长极。园区建设取得新进展。中山（河源）产业转移园三期征地顺利推进；手机生产基地加快建设，年产手机达1400万台，增长98%；国家通讯终端产品质量监督检验中心、高新区科技企业孵化器建成使用，高新技术创业服务中心正式动工建设，建立了多个产学研战略联盟。深圳（河源）产业转移园整合认定工作加快推进。“一区六园”开发面积比上年扩大7.7平方公里，基础设施和配套服务不断完善，和平钟表、紫金光学眼镜、东源新型材料、连平绿色食品加工、源城和龙川电子电器制造等主导产业加快培育发展。招商引资实现新突破。全年新签约进园区项目56个，投资总额395亿元；新开工项目92个，投资总额367亿元；竣工项目85个，投资总额53亿元。其中围绕培育“四新”产业，先后引进了北京汉能太阳能薄膜电池、广东国华新能源太阳能电站、深圳中兴通讯生产研发基地、日本东芝（TMD）TFT-LCD液晶面板、东江商贸物流城和东江源温泉度假区等大项目。万绿湖直饮水工程项目通过省发改委组织的专家评审。河源电厂一期工程如期建成发电。转移就业取得新成效。加大农村劳动力培训投入，完善就业信息服务网络，积极组织各类企业用工招聘会，落实农村劳动力到本市工业园区就业优惠政策。城镇新增就业岗位4.4万个，培训农村劳动力4.6万人，其中培训后转移就业3.7万人。全市产业转移园接纳本地劳动力7.4万人。

——宜居城乡建设成效明显。创建国家卫生城市、国家环保模范城市取得阶段性重大成果，其中“创卫”成功通过省考核验收，人居环境明显优化，城市形象明显提升。城乡规划进一步完善。在全市开展创建宜居村镇“六个一”工程活动。在全省率先按“三规合一”要求，完成了至2020年城市总体规划、城乡总体规划、土地利用总体规划等草案的修编和市区绿地系统、排水系统、环境卫生、干线路网等专项规划。开展了“公园城市”、“万绿水城”规划编制工作，完善了市区控制性详细规划。完成了全市公路网和公路运输站场等规划修编。顺利完成了紫金、龙川县城行政区划调整。基础设施进一步提升。大广高速公路连平段动工建设，汕湛高速公路河紫段开工在即，粤湘、汕昆和市区东环高速公路规划建设加快推进。惠河城际轨道项目前期工作全面展开。龙川至汕尾铁路项目纳入铁道部2010年储备项目。环市路龙川至紫金段改造顺利推进。市区河源大道二期、龙川东江大桥扩建、紫金古竹东江大桥改造工程竣工通车。市区迎客大道、万绿湖大道、西环路一期、滨江大道一期、东江西路等5条市政重点道路和珠河大桥加宽改造工程全面动工。完成国省道改造和大修路面113.4公里、县乡公路大修路面100.5公里。建成 “1·8·28”输变电工程项目22个，建成110千伏以上输电线路447千米，电网和农网改造工程年度计划全面完成。城市管理进一步规范。在全市开展了交通安全大排查大整治行动，交通秩序明显好转。加大了对城市“六乱”的集中整治和管理力度。规范了市区户外广告管理。完成了东埔河整治首期工程。对全市规模养猪场、医疗废物、工业危险物、东江干流市区段水面非法经营饮食船只、重点旅游线路沿线酒楼餐馆环境违法行为、市区噪声污染、市区五金加工场所和市区废品回收市场等开展专项整治，取得明显成效。生态环保进一步加强。在全省率先开展并基本完成林业体制改革，自筹资金分流安置林业系统富余员工近5000人。在全省率先实施全市范围封山育林，完成造林作业面积12.5万亩。市区七寨生活垃圾卫生填埋场投入试运营。市区生活污水处理厂、城南污水处理厂配套工程完成并正常运行。完成“一县一厂”污水处理厂主体工程，部分进入试运行。严厉打击矿产资源违法开采行为，受到国家“整规”领导小组通报表彰。淘汰落后钢铁产能133.5万吨、小水泥20万吨，全面完成省下达的节能减排任务，被评为“中国节能减排二十佳城市”。全市江河水质常年保持国家地表水Ⅰ—Ⅱ类标准，空气质量保持一级水平，森林覆盖率达70.8%。紫金县创建“全省林业生态县”通过省验收。

——新农村建设扎实推进。全市农林牧渔业总产值86.4亿元，增长5.4%。全面落实强农惠农政策，安排种粮、农机等补贴1.5亿元。农村年人均纯收入1500元以下的贫困户和贫困人口减少到6.3万户、20.4万人，分别下降5.3%和6.4%。生态农业稳步发展。在全省率先出台了现代生态畜牧业发展总体规划。河源现代农业科技示范园被国家认定为全国农产品加工创业基地。争取国家、省安排资金7500多万元，全面实施东江上游特色水果产业带项目建设，新建水果基地5.5万亩、油茶基地3600亩。大力推进专业镇、农业标准化建设，新增省级专业镇1个，建立农业标准化示范区28个，省和市级农业龙头企业分别增加2家和13家。粮食工作考评成绩位列全省第6位。有效监控预防高致病性禽流感等重大动物疫情工作，未发生重大动物疫情。农村基础设施不断改善。新增新农村建设示范点108个。投入城乡水利防灾减灾资金6.1亿元，新建和加固市、县城市防洪堤123公里。完成中型水库加固工程2宗、在建中小型水库除险加固工程124宗、省人大农村机电排灌议案工程211宗。在全省率先开展中小流域治理并完成规划编制工作。气象预报预警服务体系进一步完善。扶贫工作扎实推进。落实水库移民后扶政策，安排2.46亿元，扶持移民完善基础设施和发展生产，扶助符合条件的移民100%参加合作医疗保险，并在市高新区富民工业园C区建成移民生产就业新基地。扶贫开发“双到”工作扎实推进，落实扶持资金5100多万元。竞得省农村危房改造竞争性扶持资金1800万元。“千村脱困”政策全面落实，1117个贫困村全年共获分红款2601万元。

——生态旅游发展步伐加快。全年接待入市游客840.4万人次，旅游总收入35.8亿元，分别增长16.2%和15.5%。被评为“中国十大特色休闲城市”。加强了旅游设施建设。按照创建省生态旅游示范区的目标，开展“万绿生态旅游度假区”策划，推进旅游业转型升级。御临门温泉度假村被评为国家4A级旅游景区和“中国十大温泉养生基地”。按五星级标准建设的万绿湖国际会议度假中心施工进展顺利，新引进的五星级希尔顿大酒店正式动工，市区和各县城按星级标准改造新建了一批酒店。加强了景区景点管理和旅游从业人员培训，旅游服务质量不断提高。加强了旅游推介工作。成功举办第四届广东（河源）国际温泉旅游节暨第六届河源市客家文化旅游节系列活动、“唱响广东”河源赛区活动，组织旅游企业参加旅游促销系列活动，“客家古邑、万绿河源、温泉之都、恐龙故乡”的旅游品牌进一步打响。同时，房地产、商贸流通、运输仓储、连锁经营、物流配送、食宿餐饮以及信息中介、社区服务等服务业进一步发展。全社会消费品零售总额142.6亿元，增长15%。

——财税金融工作富有成效。在财税增长难度极大、资金严重短缺、建设任务繁重、民生支出加大、刚性支出剧增的情况下，注重统筹谋划、开源节流、科学理财、精心调度，为保增长促发展提供了有力的资金支持。保运转成效明显。实施科学化、精细化管理，加强了对重点行业、重点税源企业和重点税种的监管，进一步做好城市土地储备及经营，强化财政资金使用的监督管理。加大了对县区财政的专项补助，市本级对县区补助达2.79亿元，增长29%。五县一区及市本级均实现了财政收支平衡。保增长重点突出。通过搭建做实融资平台，确保了市区6条市政重点道路、客家文化公园、河源理工学校一期、市高新区三期等重点项目顺利实施，并有力支持了县区的融资工作。用足用好上级政策，积极争取上级资金扶持，全年争取中央、省级资金31.5亿元，比上年增加2.86亿元。全面落实“暖企”措施，市本级安排扶持资金3330万元，为中小企业减少行政性收费7600多万元，争取上级扶持资金3108万元。全力支持扩大消费，兑付家电、汽车、摩托车下乡补贴1900万元。金融机构支持企业融资力度加大，对中小企业授信金额达94.2亿元，发放贷款82.5亿元，增长110.3%。全市金融机构各项存款余额409.6亿元，贷款余额294.3亿元，分别增长22.4%和46.2%，存贷比达71.9%。实现了市属国有资产保值增值和劣势企业退出市场的目标。成立了4家小额贷款公司，圆满完成4家城市信用社退市工作。保民生成绩显著。全年民生投入达35.3亿元，增长16.1%。“十项惠民工程”投入27.7亿元，其中“十件实事”投入20.6亿元。同时，筹集7.7亿元偿还历史债务和解决历史遗留问题。

——社会各项事业显著进步。全面加快以改善民生为重点的社会事业发展，人民群众幸福感进一步提升。“十件实事”和“十项惠民工程”年度任务全面完成。河源理工学校首期工程、河职院实训楼和综合楼、市技工学校三期工程已建成，市委党校教研楼正加紧建设。东江教育城初具规模，在校生达2.74万人。全市新增高中阶段学位1.3万多个，高中阶段教育毛入学率达75.1%，提高5.2个百分点。在全省率先实现县域内教师工资福利与公务员“两相当”。解决了全市1.7万多名困难企业退休人员医保、部分退伍军人社保和国企早期离岗人员养老保险等历史遗留问题。城镇职工参加养老、失业、医疗、工伤、生育保险人数分别增长5%、5%、11%、5%、21%，增幅创历史新高。启动东源县新农保试点工作。全市低保救济面达7%。农村合作医疗参保率达95%，报销封顶线提高到5万元。市人民医院新院和市妇幼保健院新院建成使用，市中医院新院改建工程进展顺利，完成10所、启动12所乡镇卫生院标准化建设。加强重大疾病和公共卫生安全防控，有效做好甲流等重大疫情预防治疗工作。市博物馆、市职工文化活动中心主体工程完工，河源广播电视中心投入使用并获“鲁班奖”。顺利通过广东省二类城市语言文字工作评估。创建了“河源网”和“古邑客家”电子杂志，组建了市歌舞团和合唱团，认真开展文物普查工作。连平县城被评为“全国文明县城”。人口计生“两无”单位明显增加，三类地区基本转化为二类，计划生育率达92.81%，全面完成省下达指标。市区农贸市场建设和改造升级全面启动，其中永安市场建成使用，永和、公园、卫星等市场完成改造。市区城南（一级）汽车客运站建设前期工作正式启动。“水浸街”、“断头路”改造取得阶段性明显成效。新建行政村候车亭400个，新增村道硬底化600公里，通行政村道路100%实现硬底化。建成263宗农村饮水安全工程，解决了30万人饮水不安全问题。新增120个“农村清洁工程”试点村。全面完成农村贫困户危旧房和移民住房改造年度任务。社区办公用房建设任务基本完成。客家文化公园征地拆迁工作全面展开，笔架山公园收回政府管理并即将启动提升改造工程，市区新增绿地2.2万平方米。新建经济适用住房767套，新增廉租房503套，实现了提高市直机关企事业单位离退休人员基本养老金10%的目标。农村劳动力培训转移就业完成任务的138%。产品质量和食品安全水平进一步提高。社会各项事业不断进步。世界客属第23届恳亲大会各项筹备工作有条不紊进行。基本完成我市第二次全国经济普查和农业普查工作任务。申报国家、省级科技项目152项，争取上级科技项目资金2800多万元。扎实开展“安全生产年”和“应急管理基础建设年”活动，生产安全事故发生起数下降4.2%。深入开展社会治安综合治理和严厉打击违法犯罪专项行动，刑事案件破案率达73.4%，治安案件查处率达98.8%，其中“4·10”拐卖儿童案的侦破受到公安部表彰。认真落实信访积案化解工作，信访和越级上访人数和批次明显下降。积极推进“六好”平安和谐社区建设，源城区东埔丰源社区成功创建全国和谐社区示范区。参加全运会实现了金牌零的突破，市激流回旋基地被评为国家高水平体育后备人才培训基地。加强人才引进，规范公务员和市直事业单位财拨人员的考录管理。市人防应急指挥中心建成使用。双拥、信访、打私、人民调解、法制宣教和法律援助工作有效推进。加强了民族宗教、外事侨务、人事编制、知识产权、统计、物价、供销、信息化和对台工作，科普、档案、史志、体育、妇女、儿童、老龄、残疾人和民兵预备役等社会各项工作取得新成绩。

——政府自身建设得到加强。市政府在全省地级以上市政府绩效考核的排名居第11位，比上年前移1位，居全省东西北地区前列。督查问责力度进一步加大。建立健全作风建设明查暗访制度，完善了重点工作督查机制。相继出台了全市道路客运交通安全、市区交通秩序管理、市区市容环境卫生管理、市区建成区和未征地违法违章建筑清理整顿、市区出租车管理、森林防火工作、“创模”、“创卫”、扶贫开发“双到”工作、河源电厂建设、县区污水处理厂建设等13个问责制，有力确保了各项重点工作扎实推进。工作运行机制进一步创新。基本完成新一轮市直政府机构改革，市直议事协调机构精简55%。启动了第四轮行政审批制度改革。电子政务建设继续深化。进一步理顺市与区财政、环卫、教育、医疗卫生、文化市场、流动人口、土地储备等管理体制。加强应急管理，应对突发事件能力不断提高。廉政建设进一步加强。全面落实廉政建设责任制，进一步加强惩治和预防腐败体系制度建设，切实抓好工程招投标、土地出让、产权交易、矿权管理、规划审批、政府采购等重点领域的反腐保廉工作，对全市128个项目的财政资金使用和20个县处级单位一把手的经济责任进行了审计，社会各界对市政府领导班子党风廉政建设的总满意度排名全省第5位。依法行政工作进一步推进。编制了依法行政第二个五年规划，成立了市政府法律顾问室，建立了市政府常务会议学法、规范性文件管理、规范行政处罚自由裁量权等制度。市政府在全省地级以上市和省直单位政务公开工作考核中被评为10个优秀单位之一。接受监督自觉性进一步提高。坚持向市人大及其常委会报告工作和向市政协通报情况制度，主动接受人大法律监督、工作监督和政协民主监督，全年办理市人大代表建议81件、市政协提案126件，按时办结率和满意、基本满意率均达100%。

我们也清醒地看到，我市经济社会发展和政府工作中还存在不少困难和问题。主要是：经济基础仍然薄弱，经济总量小、财政自给率低等问题仍然突出；优化产业结构和转变发展方式任重道远；新上项目特别是新投产项目不多，拉动作用有待增强；县域经济发展后劲不足；节能降耗压力增大，生态建设和环境保护任务繁重；改善民生任务艰巨，农民增收难度增大，一些事关人民群众切身利益的突出问题有待进一步解决；一些单位和干部作风不够扎实，政府执行力有待提高等等。对这些困难和问题，我们将继续采取有效措施，认真加以解决。

各位代表，过去的一年，我们在应对国际金融危机冲击、保持经济平稳较快增长中经受住了考验，成绩来之不易。这是省委、省政府和市委正确领导的结果，是市人大、市政协和社会各界监督支持的结果，是全市人民共同努力的结果，也与深圳、中山等兄弟市和省直挂钩帮扶单位的大力帮助密不可分。在此，我代表市政府，向全市广大干部群众，向各位人大代表和政协委员，向长期关心支持我市发展的各级领导和各界朋友，向积极参与河源建设的海内外客商和外来务工人员，向驻河源解放军和武警、消防官兵，向各民主党派、人民团体、社会各界人士，表示衷心感谢和崇高敬意！
